# Measuring daily-life fear perception change: A computational study in the context of COVID-19

**DOI:** 10.1371/journal.pone.0278322

**Published:** 2022-12-22

**Authors:** Yuchen Chai, Juan Palacios, Jianghao Wang, Yichun Fan, Siqi Zheng

**Affiliations:** 1 Department of Urban Studies and Planning, Massachusetts Institute of Technology, Cambridge, MA, United States of America; 2 Institute of Geographic Sciences and Natural Resources Research, Chinese Academy of Sciences, Beijing, China; Tokyo Institute of Technology: Tokyo Kogyo Daigaku, JAPAN

## Abstract

COVID-19, as a global health crisis, has triggered the fear emotion with unprecedented intensity. Besides the fear of getting infected, the outbreak of COVID-19 also created significant disruptions in people’s daily life and thus evoked intensive psychological responses indirect to COVID-19 infections. In this study, we construct a panel expressed fear database tracking the universe of social media posts (16 million) generated by 536 thousand individuals between January 1st, 2019 and August 31st, 2020 in China. We employ deep learning techniques to detect expressions of fear emotion within each post, and then apply topic model to extract the major topics of fear expressions in our sample during the COVID-19 pandemic. Our unique database includes a comprehensive list of topics, not being limited to post centering around COVID-19. Based on this database, we find that sleep disorders (“nightmare” and “insomnia”) take up the largest share of fear-labeled posts in the pre-pandemic period (January 2019-December 2019), and significantly increase during the COVID-19. We identify health and work-related concerns are the two major sources of non-COVID fear during the pandemic period. We also detect gender differences, with females having higher fear towards health topics and males towards monetary concerns. Our research shows how applying fear detection and topic modeling techniques on posts unrelated to COVID-19 can provide additional policy value in discerning broader societal concerns during this COVID-19 crisis.

## Introduction

Fear is one of the six basic emotions [1], which is commonly considered to be a brief episode of response to a given threat, either physical or psychological [2,3]. Fear is not merely generated by the direct exposure to a threat to oneself [4]. It can also be transmitted indirectly through social transmission [5]. The perception of fear influences the decision-making process [4] and ultimately translates into behavioral change to help individuals avoid or confront the threat [6–8]. However, besides its benefits, fear could have negative mental health consequences [9] and lead to chaos in society. For instance, panic buying is a typical response to the uncertainty of crises, which depletes public resources rapidly and unnecessarily [10]. In other cases, the emotion of fear evoked by the social and political environment may lead to violence and protests [11]. Against this background, it is crucial for policy-makers to understand the causes and development of fear to identify the problems and mitigate public anxiety [2]. Such knowledge is particularly relevant for crises like COVID-19, when fear emotion rises to unprecedented intensity [12–14].

Researchers and policy-makers mainly rely on surveys to measure fear perception [15]. However, surveys have their limitations, such as limited scalability, potential sample bias, high cost, and significant time delays [16]. These drawbacks are especially prominent in the context of COVID-19 when the public sentiment evolved rapidly, and timely interventions are critical to save lives. When coupled with machine learning techniques, social media platforms can serve as a valuable tool, which enables the monitoring of public emotions and concerns with high temporal and spatial granularity. For example, using social media posts, Dodds et al. [17] explored the temporal pattern of emotions for 63 million users non-invasively; Mitchell et al. [18] estimated geographical happiness distribution using the geotagged Twitter. A recent study also shows the high correlation between social media expressed emotion measurement and traditional surveys [19], supporting the validity of such NLP methods to measure emotion.

In this paper, we study how the expression of fear for different aspects of people’s daily life changed during the COVID-19 (the contents that people posted not directly mentioning virus-related words) using social media posts and NLP. We compile an individual-based panel social media dataset containing all original posts (16 million posts) from a cohort of 536 thousand individuals from one year before the pandemic (i.e., January 1^st^, 2019) and comprehensively covers all topics, rather than restricting our sample to posts talking explicitly about COVID-19. This data allows us to control for the historical pre-pandemic fear expression patterns of individuals within our sample, and avoid the confounding effects from sample composition change (i.e., people who never posted on social media starting to post during the pandemic). Based on the compiled panel social media posts, we use the Bidirectional Encoder Representations from Transformers (BERT) model to detect fear expressions in all posts in the sample, and apply the BERTopic to extract topics in posts that expressed fear emotions.

Previous studies have conducted emotion and topic analyses on social media posts to understand public responses towards the pandemic. In particular, researchers have used machine learning or dictionary-based algorithms to monitor the trends of different emotions during the COVID-19 pandemic either using general posts [20,21] or based on COVID-19 related posts (posts containing specific keywords related to COVID-19) [22,23]. There are also emerging studies applying sentiment analysis to track the alterations in emotional well-being during the pandemic [24,25]. On the other hand, previous studies conduct topic analysis on tweets related to COVID-19 [26–28] to understand public discourse of the pandemic. Finally, another related set of papers combines emotion and topic modeling together to examine emotions reflected in social media discourse [29].

We contribute to the existing literature in two ways. First, we focus on posts not directly related to COVID-19, in order to understand the broader social impacts of the pandemic on people’s daily life. Although solely focusing on tweets talking about COVID-19 or lockdown could provide important insights into the public attitude towards the pandemic and social distancing policy, it might under-estimate the general social consequences of life disruption and depressed well-being. Therefore, our study can effectively complement existing literature by adding in this new dimension of social cost. Second, instead of modeling the trend of general emotions, we particularly focus on posts expressing high degree of fear emotion by applying topic modeling to detect public concerns reflected in the fear posts. This approach enables a low-cost instrument to understand the dynamics of the most salient negative emotion during the pandemic, which has specific policy value in detecting public concerns and supporting tailored interventions.

## Methods

### Data collection and preprocessing

We collect our social media data via the Sina-Weibo’s (the largest microblogging social media platform in China) application programming interface (API). The data contains 16 million original posts from a cohort of 536 thousand active users between January 1^st^, 2019, and August 31^st^, 2020.

Besides the raw content, we collect the exact posting time, number of likes, and re-posts for each post. To ensure data quality, we follow several rules when collecting data and constructing the research database: 1) We only collect posts from those users who registered before January 1^st^, 2019; 2) We exclude the posts generated by institutional accounts (e.g., companies and organizations) from our sample; 3) We drop users with post numbers within the top 10% to reduce the influence from extreme posters; 4) We randomly select and scrutinize 50 thousand posts to identify advertisements with a fixed format (For instance: “I am the 3545th to celebrate the shopping festival, please join us!”). We then apply regular expressions to remove advertisements in these formats for all posts; 5) We apply a series of functions to remove URLs, emojis, special characters, hash symbols from the posts to reduce the impacts of irrelevant information.

**Fig 1:**
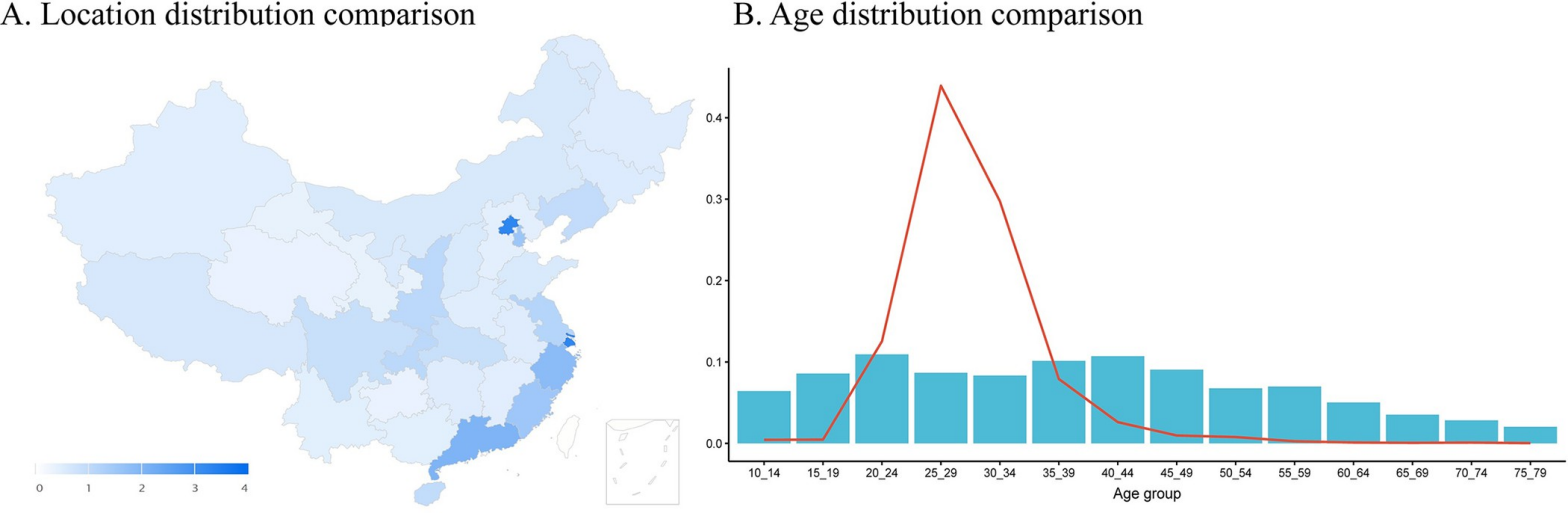
Comparison between Weibo user and Chinese 2010 census. Panel A (left) shows the oversampling rate for provinces in China. A more saturated color represents the larger ratio of relative proportion for each province of Weibo users comparing to that of the 2010 census data. Beijing (oversampling rate 6.53) and Shanghai (oversampling rate = 3.25) are the two most oversampled provinces, followed by provinces in the east of China. Panel B (right) shows the age distribution comparison. Blue bars represent census while red line depict Weibo users. Weibo users are more concentrated at a younger age range between 20–40, indicating the disproportionate distribution.

In addition, we retrieve all the publicly accessible personal information from the profile page of each individual in our sample, including the birth date, gender, number of fans, number of followers, and the registration location. Table 1 shows the summary statistics of our final sample. All people provide gender information, with 65.31% users reported to be a woman. In total, 63.0% users provide birth date, with average age of 29.01 (SD = 5.85, Min = 10, Max = 80). Compared to the Chinese 2010 demographic census, our users are more concentrated in bigger, and coast cities and are younger (Fig 1A and 1B).

**Table 1:** Collected Weibo user statistical information.

Variable	Observations	Mean	St. Dev	Min	Max
**Age**	337,579	29.01	5.85	10	81
**Female (1 = Yes)**	536,153	0.65	0.48	0	1
**Number of fans**	536,153	4,403	149288	0	56,407,118
**Number of followers**	536,153	4,02	477	0	20,000

### Expressed fear emotion classification using natural language processing

Natural Language Processing (NLP) is a computational method that translates unstructured large-scale text data into structured measures [30]. Sentiment analysis, a sub-area of NLP, is purposefully designed to evaluate the emotional status embedded in the text [31]. An increasing number of studies attempt to detect the change of perceptions or attitudes on social media either towards general or specific topics based on the measures generated from these methods [32].

In this study, we use BERT, a text classification model developed by Google [33], to classify each post into six categories of emotions (i.e., Anger, Fear, Happiness, Sadness, Surprise, and Others). Specifically, we finetune a pre-trained BERT model provided by [34] using our data and then impute the likelihood of expressing emotion in each post for each of the six emotions. The posts are tagged with the emotion of the highest possibility.

Following Lyu et al. [22], we constructed a multi-class emotion dataset to train the BERT model that consists of the following three parts, including Natural Language Processing and Chinese Computing (NLPCC) emotion analysis dataset (45 thousand sentences), the Evaluation of Weibo Emotion Classification Technology of Social Media Processing 2020 (SMP2020-EWECT) (40 thousand sentences), and a self-constructed dataset that labelled 3 thousand extra posts following the same protocol as in the other two databases. The first two are publicly available datasets, which NLPCC was constructed in 2014 and SMP2020 was constructed in 2020 during the COVID-19 period. Given that people might have fundamental changes in expressing emotions compared to the pre-pandemic period, the host of SMP2020 divided the posts into two general topics, i.e., non-COVID-19 topics and COVID-19 topics. Sentences in non-COVID-19 topic category are covering a wide range of daily life topics such as reading books, having meals etc.; while sentences in COVID-19 topic category are collected by searching COVID-19 related keywords, which are normally centering around the information of COVID-19, reporting cases and news etc., We believe including COVID-19 topics is of importance as it could help the model to rule out the bias introduced by keywords such as “virus”.

**Fig 2:**
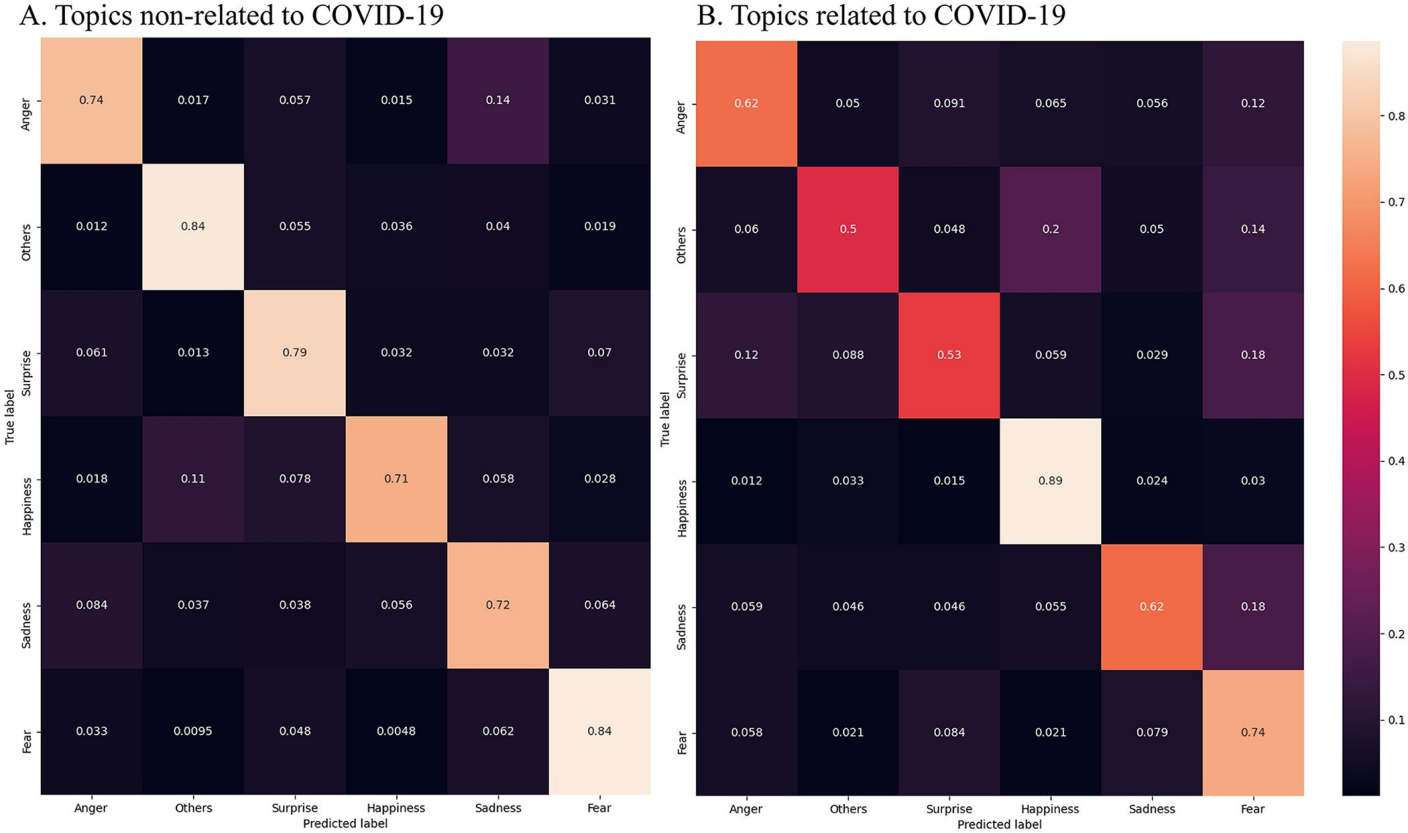
Confusion matrixes of the model performance. The figures display the performance of the deep learning model of detecting each of the six emotions considered in the study. Panel A (left) shows the proportions of posts correctly classified in topics that do not relate to COVID-19, and Panel B (right) displays the performance in topics that relate to the COVID-19 pandemic.

In total, after combining three sources of datasets as one and ensure the class balance, we have 3,719 for each of the six emotions. We assign 80% of the posts to the training dataset, and 20% to the validation and additional eight thousand posts in two general topics provided by SMP-2020 as the test set, we train a one-layer fully connected network to achieve emotion classification. Overall, the model achieves 74.43% accuracy on the validation dataset. On the testing datasets, the overall accuracy is 75.84% and 74.00% for non-COVID-19 topics and COVID-19 topics respectively. For fear emotion, the model gets 84% and 74% for two topics (Fig 2).

To better understand how the fear in topics not directly related to COVID-19 developed, we construct a dictionary of COVID-19 related words (Table 2). The post that contains any word in the list will be treated as COVID-19 related posts. S1 Fig in S1 File shows how fear posts classified as COVID-19 and non-COVID-19 related evolved on a daily basis. For the construction of this word list, please refer to S1 File section.

**Table 2:** COVID-19-related words and share of mentions.

Index	Word	Translation	Share of posts in 2019	Share of posts in 2020
1	疫情	pandemic	0.000%	1.083%
2	防控	control	0.002%	0.051%
3	新型	new type	0.005%	0.061%
4	冠状	corona	0.000%	0.055%
5	病毒	virus	0.016%	0.194%
6	爆发	break out	0.004%	0.051%
7	肺炎	pneumonia	0.005%	0.127%
8	医护	medical care	0.004%	0.056%
9	抗疫	anti-virus	0.000%	0.049%
10	口罩	mask	0.018%	0.452%
11	感染	infection	0.028%	0.078%
12	N95	N95	0.000%	0.012%
13	隔离	isolation	0.020%	0.184%
14	确诊	confirmed case	0.005%	0.059%
15	疑似	suspected	0.007%	0.024%
16	病例	case	0.005%	0.040%
17	告急	in danger	0.002%	0.006%
18	疫苗	vaccine	0.008%	0.037%
19	封城	lockdown	0.000%	0.039%
20	解封	lift restriction	0.001%	0.028%
21	湖北	hubei	0.020%	0.087%
22	武汉	wuhan	0.142%	0.576%
23	核酸	nucleic acid	0.000%	0.030%

### Topic modeling

To understand why people express fear in social media during a health crisis, we implement a topic modelling algorithm to discover the abstract topics within the posts in the dataset. Topic modelling is widely used by researchers to understand public opinion [27,35]. BERTopic, a state-of-the-art machine learning method that leverages BERT embeddings, uniform manifold approximation and projection (UMAP) dimensionality reduction, hierarchical density-based spatial clustering of applications with noise (HDBSCAN), and class-based term frequency-inverse document frequency (c-TF-IDF) [31] to identify interpretable topics. Using a pre-trained multi-lingual sentence embedding model to encode the text, we apply BERTopic on non-COVID-19 fear posts to identify the fear sources in people’s daily life. We apply the model on COVID-19 posts as well to support the analysis. To decide the best topic size, we impute the coherence score by varying the number of clusters and select topic sizes as 60 and 30 (S2 Fig in S1 File).

To visualize the most informative keywords for each topic, we take the following steps: (1) We apply BERTopic model on the vectorized sentences and get the class id for each of the sentence. (2) We join the sentences in the same topic class together and apply class-based TF-IDF algorithms to extract the TF-IDF value for each word. (3) We select the top 3 Chinese words with the highest TF-IDF score within each class and translate them into English using Google translate.

## Results

### General trend of fear posts

**Fig 3:**
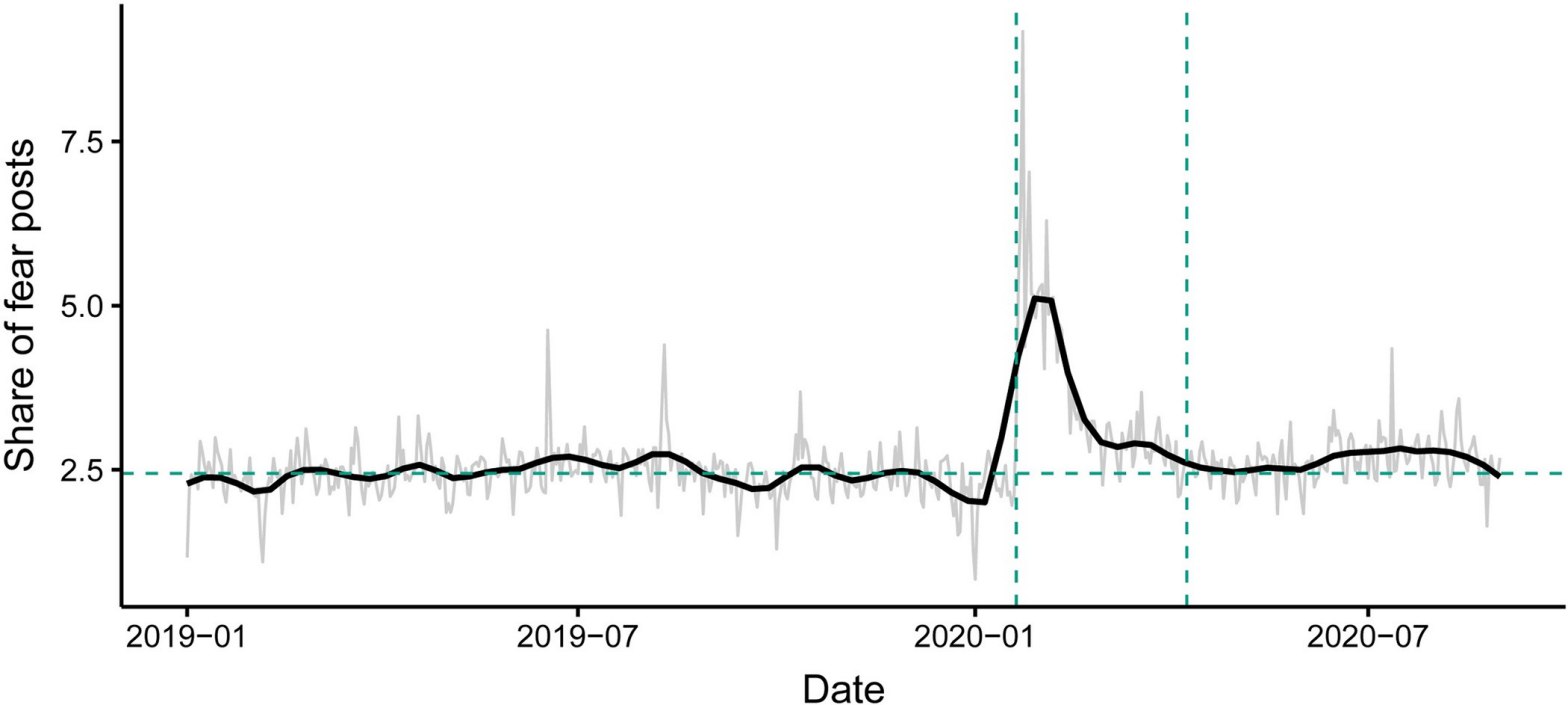
Daily share of posts containing fear emotion. Line graph shows the daily trend of the share of fear posts among all posts. Light grey and the dark black line show the original and smoothed time series respectively. To better locate the peak COVID-19 period, we draw two vertical dashed lines in the plot showing the start of COVID-19 (left, January 20th) and the re-open date of Wuhan city (right, April 8th). The horizontal dashed line depicts the average share of posts during the year 2019.

The trained emotion classification model identifies 381K fear posts (203,497 fear posts in 2019 and 178,123 fear posts from January to August, 2020) in total from the original 16 million Weibo posts. Fig 3 shows the daily share of posts expressing fear over the total number of posts. The results show that the frequency of fear expressions is relatively stable across 2019, with 2.45% posts (600 posts) on average classified as fear posts (i.e., posts dominated by fear emotion) every day. In 2020, the share of posts labelled as fear expressions reaches a peak of 9.1% (1,868 posts) on January 23^rd^ (the date that epi-center Wuhan city was announced to lockdown). The share of fear posts steadily drops afterwards and remains stable around 2.64% (681 posts) of total posts after April 8^th^, 2020, slightly higher than the 2019 baseline. Besides the onset of COVID-19, there are several spikes in the fear posts within our sample period, which are mostly affected by weather and catastrophes (e.g., Typhoon Lekima elicits a 7.64 SD spike; Hebei earthquake generates a 7.17 SD spike; as a reference, Wuhan lockdown has an 18.60 SD spike) (see S4 Table in S1 File)

### Evolution of non-COVID-19 related fear topics

We use BERTopic to automatically split the data into meaningful clusters. In total, there are 60 fear topics unrelated to COVID-19 (S1 and S2 Tables in S1 File, with sample posts presented in S3 Table in S1 File and cross-topic relationship in S2 Fig in S1 File). The original fear topics lie into six large categories: Health-related fear topics (38.54%) take up the largest share among all the fear posts, followed by relationship (12.10%), weather and catastrophe (10.19%), transportation (8.32%) and work/ education (6.15%). To estimate the magnitude of fear alterations associated with each topic induced by the pandemic, we conduct *t*-tests to compare the fear share by topics in different sub-periods after the peak COVID-19 pandemic with the same period in 2019. Specifically, we define the following two sub-periods in China as follows: (1) COVID-19 peak period started from January 20^th^, 2020 and ended on April 8^th^, 2020 (i.e., the date when the city of Wuhan re-opened); (2) post-COVID-19 period started from April 9^th^ and ended at August 31^st^ for 2020.

**Fig 4:**
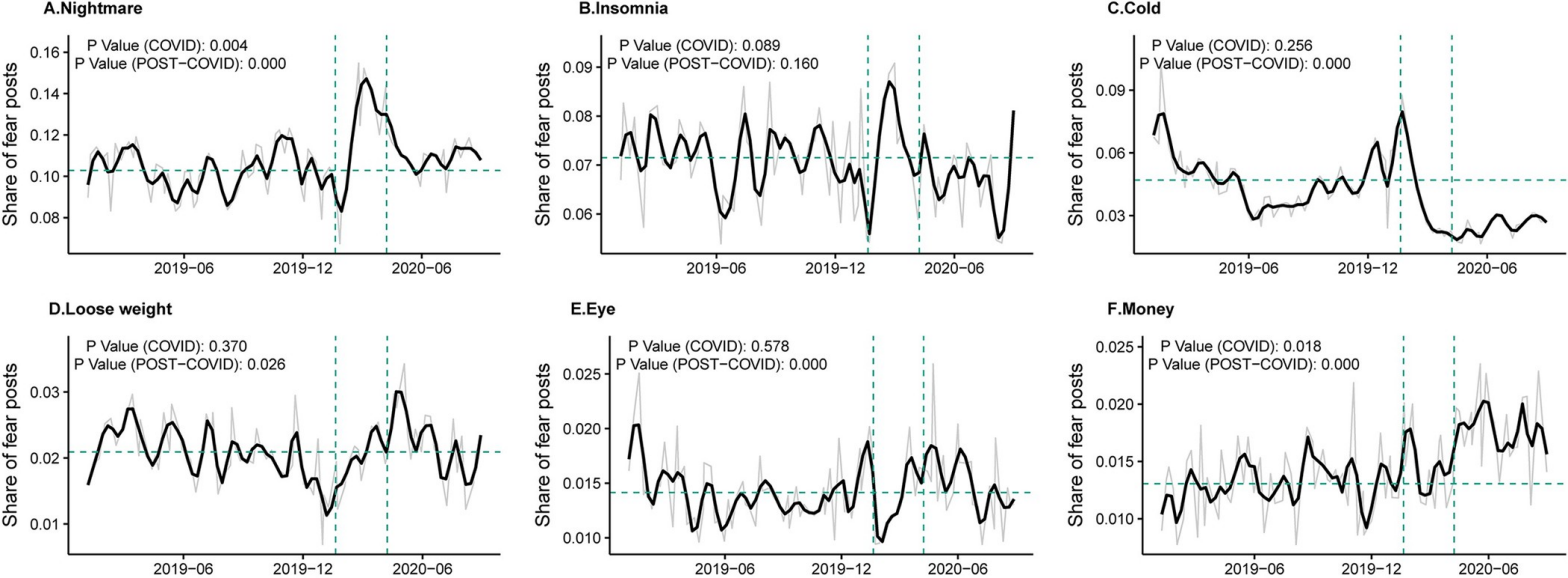
Share of fear posts by topic. Line graphs show the number of posts for six non-COVID-19 related topics by week. The name of each subplot is the most informative word for each topic. The dark solid lines in each subplot display the smoothed number of posts per day. *P*-value (COVID) and *P*-value (post-COVID) indicate the *t*-test results testing the differences of trend between 2020 peak COVID-19/ post-COVID-19 periods with the same period in 2019.

Health and work-related topics had the largest change during the COVID-19 peak sub-period (from January 20^th^, 2020 and ended on April 8^th^, 2020). In particular, we find that topics about sleep (i.e., nightmare and insomnia) have the largest share in fear posts during our sample period of two years. On average, 10% and 7% of fear posts are related to nightmares and insomnia, respectively. As shown in Fig 4A, during the COVID-19 peak period, fear posts with contents of “nightmare” significantly increased, reaching a share of 16% of all fear posts. Though this share dropped after the COVID-19 peak sub-period, it remains significantly higher than the same period in 2019 until the end of August, indicating a long-lasting impact. Since “nightmare” could be expressed not only as having an unpleasant dream but also as a way to describe a disastrous event, we further explore the posting time within a day to check whether the fear posts are likely to be sleep-related. We assume that if the “nightmare” is used to describe the awful dream, people are more likely to post in the morning right after having a bad sleep. The results in S4 Fig in S1 File indeed show that posts about “nightmare” are concentrated in the early morning, and the posting times within a day are similar in 2019 and 2020, indicating that there is no significant change in word usage. “Insomnia”, i.e., unable to sleep, displays a similar spike during the COVID-19 peak period (Fig 4B), suggesting that people had more difficulties falling asleep. The share of “insomnia” posts soon recovered to pre-pandemic status after the beginning of April. Besides sleep disorders, among health topics, we also notice a significant drop in posts mentioning “cold and fever” (Fig 4C), and a significant increase in posts mentioning “lose weight” (Fig 4D) and “eye” (Fig 4E).

Besides health, work is one of the key areas for which the COVID-19 pandemic created significant impacts. Many researchers have identified the economic impacts of COVID-19 infections and the associated policies to prevent infections [12,36]. The lockdown policy could curb the infections but at the same time prevent people from going to work. The share of posts mentioning “money” increased significantly since the beginning of the COVID-19, suggesting a rise in financial concerns in our sample. After checking the content of posts within the money topic, we find that people are paying more attention to the importance of having savings given the economic stress imposed by the pandemic.

### Gender differences

Females tend to have a higher tendency to express fear in social media (Table 3). In our sample, 19.81% of female users have posted contents containing fear during our observation period with an average of 2.48 fear posts per person, while only 12.64% of male users have posted fear content with 2.09 posts per person. The results of t-tests show that these gender differences are significant (coefficient = 0.39, P-value = 0.000). Such gender differences in fear are salient both in the absolute number of fear posts and in the share of fear posts over total tweets (S5 Fig in S1 File), suggesting that the fear differences by gender is not driven by females being more expressive.

**Table 3:** Gender statistics on fear expression.

Gender	Has posted fear	# of user	Ratio	Avg # of fear posts
Female	Yes	69,352	19.81%	2.48
Male	Yes	23,515	12.64%	2.09

**Fig 5:**
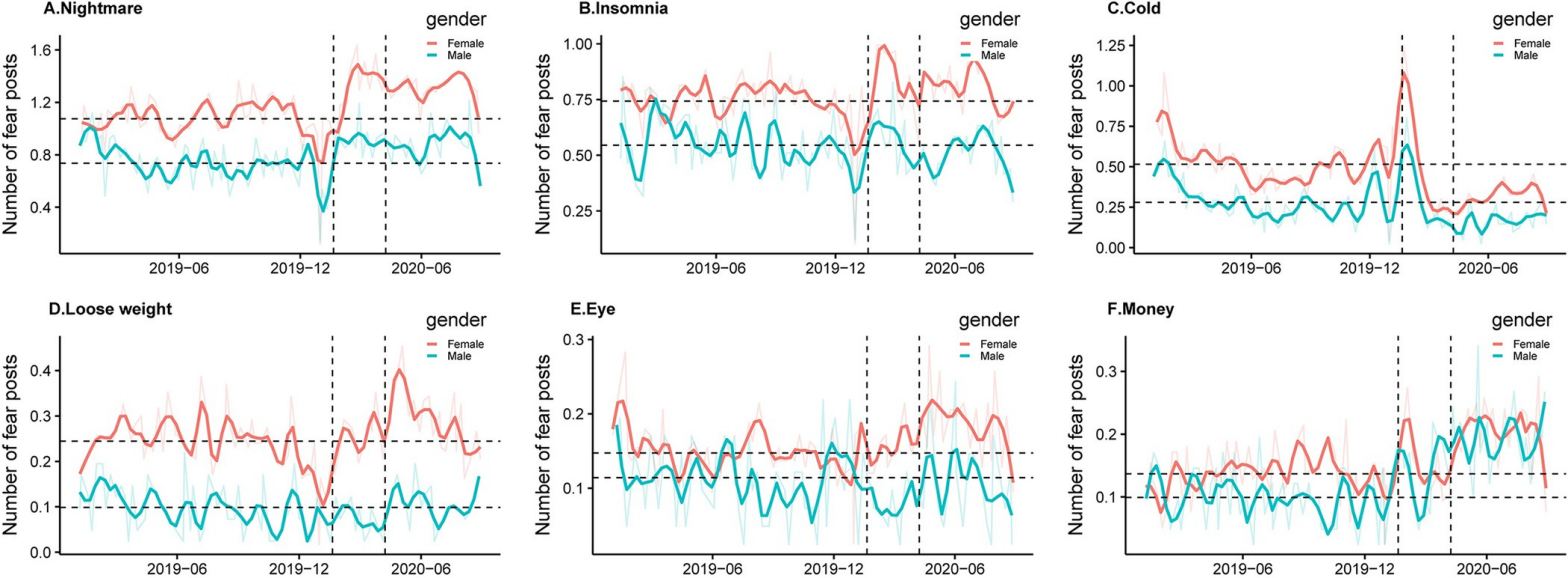
Number of fear posts by topic by gender. Line graphs show the weekly average number of fear posts generated by every 1,000 users in each gender (Female: Solid line above, Male: Solid line below). Two horizontal dashed lines depict the baselines (the mean values of 2019) by gender. Two vertical dashed lines show the start date of COVID-19 (January 20^th^) and the re-open date of Wuhan (April 8^th^).

Previous research have identified significant gender differences during the COVID-19 period in aspects such as risk perception, time use, and compliance to social distancing policies [37,38]. Here we further explore gender differences in COVID-19 induced fear by topics (Fig 5). For each topic, we use four t-tests to investigate the change in fear during and after the peak COVID-19 pandemic compared to 2019 baseline by gender (S5 Table in S1 File). The detected gender differences described below are robust when we control for the number of fear posts by gender to eliminate the concern of different expressiveness (S6 Table in S1 File).

Regarding the fear related to “nightmare”, we find that both genders increase posting during the COVID-19 period, with females having a larger and more significant extent (coefficient = 0.251, *P*-value = 0.005) comparing to males (coefficient = 0.103, *P*-value = 0.193). After the COVID-19 peak sub-period, both genders remain to have a significantly higher frequency of nightmare-related fear posts relative to their levels in 2019 (with coefficients of 0.270 and 0.197, *P*-value 0.000 and 0.000 for females and males respectively). In addition, the insomnia topic shows a similar pattern that the female had a significant increase in posting during the COVID-19 period (coefficient = 0.1, *P*-value = 0.057). The results from the two sleep-related topics suggest that females are more likely to have sleep disorders during the COVID-19 and such impact lasts for months.

**Fig 6:**
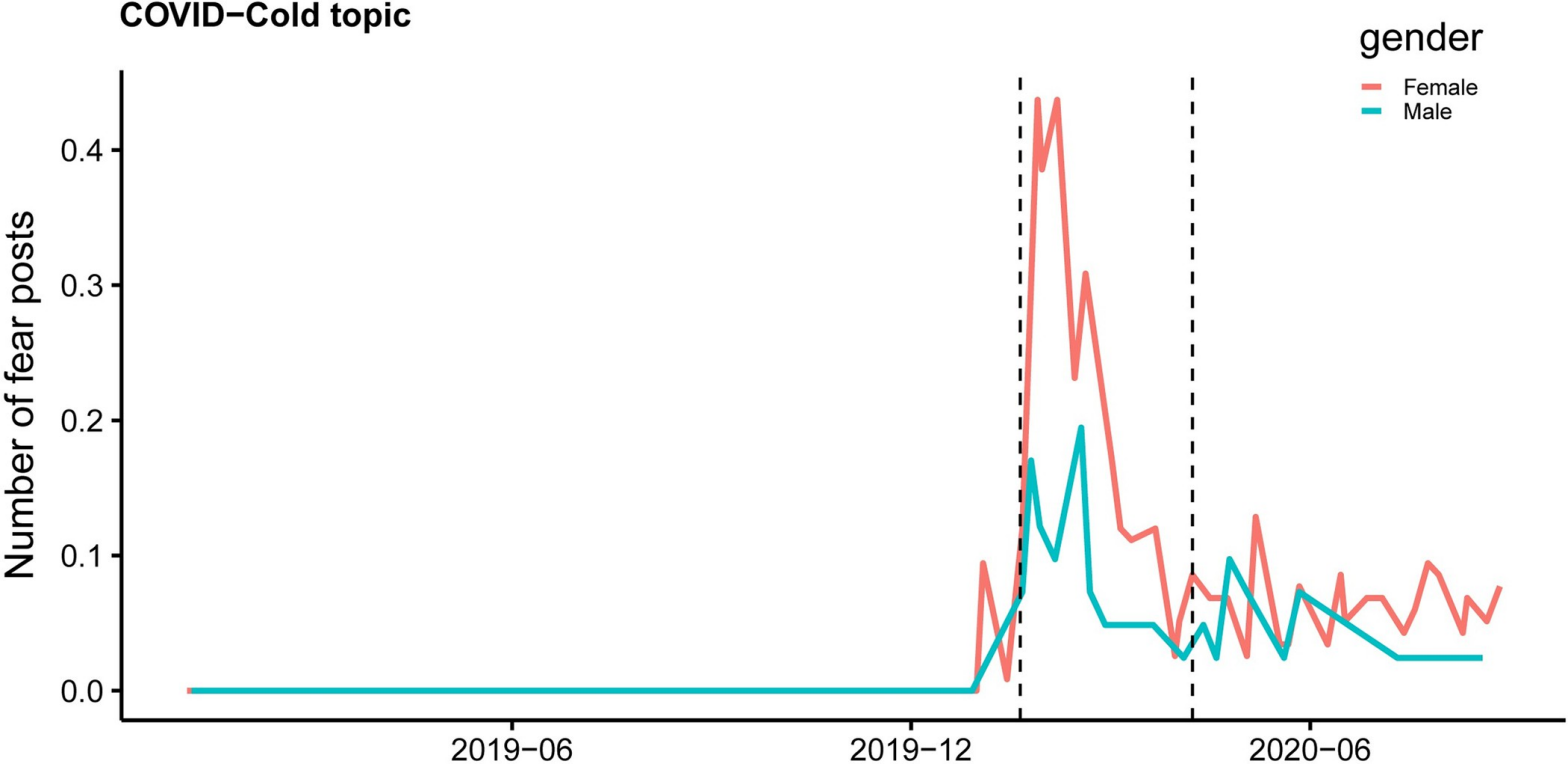
Number of COVID-19 related fear posts (cold topic) by gender. Line graphs show the average number of fear posts generated by every 1,000 users in each gender by week (Female: Solid line above, Male: Solid line below).

We also detect the differential changes by gender in the “cold and fever” topic. Cold and fever are prevalent in winter seasons, as shown by the peaks at the beginning of 2019 and 2020. However, unexpectedly, the number of non-COVID-19 posts related to cold drops quickly since the start of COVID-19 and with females reducing more than males. We conduct a difference-in-differences analysis at post-level while controlling for age and province fixed effects to quantitively examine the gender differences to mention “cold and fear” during the pandemic (S7 Table in S1 File). To understand the reason, we also investigate the topic analysis result for COVID-19 related posts. Fig 6 shows the posts associating “cold and fever” with COVID-19 by gender. Both genders have a peak after the burst of pandemic, while females are more likely to include COVID-19 related words when mentioning cold and fevers. We further check the posts’ content and discover that females are more likely to associated themselves and their family members’ cold symptoms to COVID-19 and express concerns.

Another pattern we find is related to losing weight. Males reduce the posts related to losing weight during the peak COVID-19 period and females increase the posts in this topic after the COVID-19 peak period (Fig 5D). This suggests that people in our sample were less concerned about body shape during the peak pandemic period yet soon start to pay more attention to it once they need to resume work and social activities. The increasing concerns for weight loss could also indicate a reduction in physical activity, as found in previous studies [39].

Finally, both males and females post more about monetary topics during the COVID-19 period, with males having a larger extent (coefficient = 0.042, *P*-value = 0.064) comparing to females (coefficient = 0.034, *P*-value = 0.051). Such a concern becomes more significant after the COVID-19 peak period (Male coefficient = 0.090, *P*-value = 0.000; Female coefficient = 0.062, *P*-value = 0.000). The work-related topic result indicates that, in opposite to health-related topics, males pay more attention to the economic side, indicating a different type of stress. The result could serve as a potential explanation of why men are having a higher suicide rate during the COVID-19 period [35].

## Discussion

This study shows that the COVID-19 has altered people’s fear perception towards daily life topics unrelated to virus infection, and the perception change can last for months after the peak pandemic period. We find that the daily-life fear topics in the COVID-19 period which has significant change can be best classified into three clusters: (1) symptoms of fear (such as “nightmare”, “insomnia”), (2) fear related to other health problems (such as “lose weight”, “eye”), (3) fear about socio-economic consequences (such as “money”).

Our results have important implications. First, the significant increases in fear towards these topics indicate an increase in the mental distress and anxiety caused by the COVID-19. Our result shows that fear posts related to “nightmare”, the largest non-COVID-19 related fear source, take up a significantly higher proportion of fear posts even months after the peak pandemic. Deteriorated sleep quality brought by mental distress during the COVID-19 could contribute to latent risks for the population’s physical and psychological health, which should receive added attention. Second, our results suggest that COVID-19 and related policies induced health and financial concerns. Staying at home was accompanied by a reduction in physical activities and an increase in screen time, thus inducing more fear posts for weight and eye problems. The increased attention to “money” indicates that people were also faced with higher economic burdens during the pandemic. These results reveal the importance of paying attention to the broader social consequences of the COVID-19 on people’s daily life, instead of solely focusing on the COVID-19 related posts when analyzing the fear response. Finally, our findings indicate that both genders are affected by the COVID-19 in general with different focus. Besides showing the topic trends on specific topics, we reclassify 60 posts into six general aspects including “Health”, “Weather and Catastrophe” “Transportation” etc., and visualize the temporal trend (S6 Fig in S1 File). While females are more sensitive to health or relationship issues, males are more concerned with transportation and money (a sub topic under “Work and Education”). A potential mechanism, as shown by previous literature, is that females are more concerned about childcare while males are more concerned about paid work during the pandemic [40]. Our results call for further explorations of the reasons that underlie the sub-group differences in fear responses to assist tailored policies.

Beyond the results, our method has broader applications for computational social science research. Using various data and methods, previous studies have found consistent findings to ours, such as that COVID-19 leads to sleep disorders [41,42], job insecurity and financial concerns [43], and gender differences [9,44]. However, our unique advantage is that we can use one data source to identify the most important public concerns in an unsupervised way and rank their importance. Our method also allows for real-time monitoring with high temporal and spatial granularity, a characteristic particularly important during unexpected public crises.

It is worth noting that our method also has several limitations. First, users of social media platforms might not be able to represent the whole population. Research has found that social media users are younger and are more concentrated in big cities [45] which we also observe in our sample. Second, we use the expressed fear within posts to proxy the fear emotion. Whether the expressed emotion could accurately represent the inner emotional state is still a nascent research area and thus without a clear conclusion. Third, even if the expressed fear can represent the actual feeling of users, we only observe changes in the number of posts with fear as the dominant emotion. Our algorithm does not directly measure the fear intensity of each post at the current stage. Finally, comparing to a delicately designed survey, using the data-driven method to automatically extract information from unstructured social media posts has unavoidable measurement errors, since the neural network can only capture the general knowledge from training samples and neglects the varying outliers. We hope that our work can motivate more future studies to explore the value of computational methods to understand human emotions and behaviors.

## Supporting information

S1 File Supporting information including supporting materials and methods, supporting figures, and supporting tables. (DOCX)
