# Supplementary material for: Measuring daily-life fear perception change: A computational study in the context of COVID-19
Source: PLoS One. 2022 Dec 22;17(12):e0278322. doi: 10.1371/journal.pone.0278322 (PMC9779044; doi:10.1371/journal.pone.0278322)
Supplement: S1 File — (DOCX) [file pone.0278322.s001.docx]

S1 Section: COVID-19-related word selection

To construct the list of words related to COVID-19, we take a two-step approach. First, we fetch COVID-19-related words from previous literature, and second, iteratively expand the keywords with human-in-loop to capture more comprehensive words under the unique language context of Chinese.

In particular, for step 1, we build our initial COVID-19-related word list based Wang et al., (2022) [1], which defines an exhaustive list of COVID-19-related terms. For each of the words in the list, we find the most relevant words in Chinese to form our dictionary. By searching tweets that contain any word in the dictionary, we establish our first version of COVID-19-related posts.

For step 2, we take an iterative method with human-in-loop following Hossny et al., (2018) [2]. We implemented a BERTopic analysis on these selected posts from step 1 to find the most informative words for each topic within the COVID-19-related posts. Two researchers go through the top three words of each topic and decide if the word is COVID-19 specific to be included for the next round. A third researcher is involved whenever there is any disagreement in the word list definition. We stop the word expansion when including a new word adds less than 2% posts.

The selected words after the two steps are displayed in Table 2 of the manuscript. It is important to note the differences in use of words across languages. In particular, words like “suspected” and “in danger” seem quite general in English. However, the corresponding Chinese words are very related to the COVID-19 context, which is vastly used during the pandemic. For example, “疑似” (suspected) usually follows “COVID” and is followed with “病例” (cases) in our sample of posts, inferring that a patient is likely to get COVID. In addition, our selection method by design only excludes words that are specific to the COVID-19 context. For instance, although insomnia happened a lot during the COVID-19, the word “insomnia” was equally prevalent in 2019, before the COVID-19 pandemic, suggesting it is not a COVID-19 specific symptom.

1. Wang J, Fan Y, Palacios J, Chai Y, Guetta-Jeanrenaud N, Obradovich N, et al. Global evidence of expressed sentiment alterations during the COVID-19 pandemic. Nat Hum Behav. 2022;6: 349–358.

2. Hossny AH, Mitchell L. Event Detection in Twitter: A Keyword Volume Approach. 2018 IEEE International Conference on Data Mining Workshops (ICDMW). ieeexplore.ieee.org; 2018. pp. 1200–1208.

S1 Table: COVID-19 related topics

| Topic | Size | Word 1 | Word 2 | Word 3 |
| --- | --- | --- | --- | --- |
| 1 | 4204 | epidemic | the end | past |
| 2 | 1701 | isolation | 14 | nucleic acid |
| 3 | 920 | masks | go out | naked running |
| 4 | 891 | masks | cannot buy | pharmacy |
| 5 | 875 | baby | fever | hospital |
| 6 | 753 | virus | terrible | infection |
| 7 | 677 | masks | allergy | ear |
| 8 | 532 | dream | dreamed of | in the dream |
| 9 | 515 | wuhan city | four people | hubei province |
| 10 | 508 | wuhan city | lockdown | hope |
| 11 | 457 | materials | emergency | request for help |
| 12 | 354 | united states | diagnose | covid |
| 13 | 314 | hope | past | epidemic |
| 14 | 311 | wuhan city | rainstorm | the weather |
| 15 | 303 | pneumonia | new type | covid |
| 16 | 250 | 2020 | kobe bryant | spring festival |
| 17 | 245 | epidemic | terrible | anxiety |
| 18 | 243 | subway | masks | stop |
| 19 | 216 | diagnose | grand total | case |
| 20 | 210 | cannot sleep | insomnia | go to bed |
| 21 | 207 | vaccine | rabies | nine-valent |
| 22 | 203 | aircraft | flight | airport |
| 23 | 183 | wuhan city | virus | infection |
| 24 | 182 | holiday | go to work | jobs |
| 25 | 182 | past | epidemic | faster |
| 26 | 181 | lockdown | reopen | don't understand |
| 27 | 180 | vaccine | kitty | a |
| 28 | 163 | pneumonia | wuhan city | covid |
| 29 | 156 | china | country | epidemic |
| 30 | 152 | n95 | masks | medical |

S2 Table: Non COVID-19 related topics

| Topic | Size | Word 1 | Word 2 | Word 3 |
| --- | --- | --- | --- | --- |
| 1 | 17468 | nightmare | dream | bad dream |
| 2 | 11410 | insomnia | cannot sleep | go to bed |
| 3 | 6705 | cold | fever | cough |
| 4 | 6366 | drive a car | driver | high speed |
| 5 | 5271 | afraid | lonely | terrible |
| 6 | 4935 | dizziness | height | head |
| 7 | 4732 | rain | rainstorm | heavy rain |
| 8 | 4320 | the film | terror | horror film |
| 9 | 4292 | earthquake | felt | feel |
| 10 | 3978 | weibo | circle of friends | wechat |
| 11 | 3588 | anxiety | tension | recent |
| 12 | 3393 | lose weight | body weight | terrible |
| 13 | 3063 | terrible | habit | world |
| 14 | 2986 | 2020 | 2019 | end of world |
| 15 | 2919 | dogs | dog | a |
| 16 | 2883 | wisdom teeth | tooth extraction | tooth |
| 17 | 2697 | hair | hairline | bangs |
| 18 | 2635 | teacher | start of school | school |
| 19 | 2484 | a | kitty | kitten |
| 20 | 2438 | drink | alcohol | drunk |
| 21 | 2394 | marry | fear of marriage | marriage |
| 22 | 2387 | money | some | confused |
| 23 | 2383 | save | help | child |
| 24 | 2313 | eye | eyelid | right eye |
| 25 | 2247 | subway | high speed rail | train |
| 26 | 2187 | eat | dare | terrible |
| 27 | 2054 | milk tea | coffee | cup of |
| 28 | 1974 | thunder | lightning | thunder and lightning |
| 29 | 1967 | examination | gaokao | tension |
| 30 | 1928 | go to work | not realized | jobs |
| 31 | 1901 | go out | go home | dare |
| 32 | 1888 | hospital | doctors | physical examination |
| 33 | 1880 | mosquito | mosquito coils | mosquito net |
| 34 | 1772 | guangzhou city | winter | freeze to death |
| 35 | 1689 | this year | a year | chinese new year |
| 36 | 1684 | month | july | august |
| 37 | 1680 | aircraft | airport | flight |
| 38 | 1606 | soul | hindsight | demons |
| 39 | 1588 | death | sudden death | go to die |
| 40 | 1587 | next time | na na | a bit |
| 41 | 1482 | foggy | roller coaster | climb the mountain |
| 42 | 1468 | poisonous | poisoning | mushroom |
| 43 | 1446 | nowadays | hot | tomorrow |
| 44 | 1428 | heart | heartbeat | accelerate |
| 45 | 1403 | clothes | short sleeve | long johns |
| 46 | 1395 | baby | pregnancy | doctors |
| 47 | 1376 | chongqing city | fog | hotpot |
| 48 | 1363 | elevator | power failure | stairs |
| 49 | 1307 | typhoon | windy | gale |
| 50 | 1292 | sisters | sister | brother |
| 51 | 1223 | faker | bilk | phone |
| 52 | 1210 | get away | evade | unable to escape |
| 53 | 1148 | love song | rapper | a song |
| 54 | 1145 | belly | gastroscopy | gastroenteritis |
| 55 | 1130 | blood pressure | exsanguinate | hospital |
| 56 | 1126 | age | age | afraid |
| 57 | 1106 | holiday | holiday | go to work |
| 58 | 1094 | weekend | monday | friday |
| 59 | 1092 | explosion | place | flammable |
| 60 | 1083 | 30 | thirty | 25 |

S3 Table: Example posts of identified non-COVID-19 related topics

| Topic | Topic Keyword | Content |
| --- | --- | --- |
| 1 | Nightmare | 做梦梦到领导……被骂醒了  Dreaming of the leader…woke up after being scolded |
| 1 | Nightmare | 恶梦缠身怎么治！  How to cure nightmare |
| 2 | Insomnia | 最怕夜幕降临，多少个不眠夜  I am most afraid of nightfall, so many sleepless nights. |
| 2 | Insomnia | 一直熬夜会不会猝死啊  Will I suddenly die if I stay up frequently |
| 12 | Lose weight | 最近不敢看的两个东西。体重秤还有日历  Two things that I dare not look at recently, weight and calendar |
| 12 | Lose weight | 体重噌噌噌往上涨，太可怕了  It is so horrible that weight is going up |
| 22 | Money | 世人慌慌张张，不过图碎银几两  People are panicking and hurrying around, just to earn some money |
| 22 | Money | 大灾之时，才知道余钱余粮的重要性  Only at the time of disasters that we know the importance of money and food. |
| 24 | Eye | 近视眼求检查，有点怕  I need a myopia examination, a little scary |
| 24 | Eye | 用眼过度了吗，总觉得眼睛雾蒙蒙的  Do I use my eys too much? I always feel that my eyes are foggy |

S4 Table: Abnormal fear share and its reason

| Date | Deviation to mean | Reason |
| --- | --- | --- |
| 2019-04-18 | 2.34 | Earthquake in Taiwan, China |
| 2019-04-24 | 2.00 | Release of The Avenger 4 |
| 2019-06-17 | 5.91 | Earthquake in Sichuan, China |
| 2019-06-18 | 2.54 | Earthquake in Sichuan, China |
| 2019-07-22 | 2.04 | Extreme hot day in north and storm in south |
| 2019-08-09 | 3.58 | Typhoon Lekima |
| 2019-08-10 | 7.64 | Typhoon Lekima |
| 2019-08-11 | 3.79 | Typhoon Lekima |
| 2019-10-12 | 3.64 | Earthquake in Guangxi, China |
| 2019-10-14 | 2.52 | Korean actress suicide |
| 2019-11-27 | 2.93 | China actor died while shooting a show |
| 2020-05-26 | 2.07 | Earthquake in Beijing, China |
| 2020-07-06 | 2.50 | Storm in Shanghai, China |
| 2020-07-12 | 7.17 | Earthquake in Hebei, China |

S5 Table: T-test results of number of posts by 1000 people in each gender by week.

| Topic | Gender | Period | Coefficient | SE | P-Value |
| --- | --- | --- | --- | --- | --- |
| Nightmare, Dream | Female | COVID-19 Peak | 0.251 | 0.080 | **0.005 ***** |
| Nightmare, Dream | Male | COVID-19 Peak | 0.103 | 0.077 | 0.193 |
| Nightmare, Dream | Female | Post COVID-19 | 0.270 | 0.037 | **0.000 ***** |
| Nightmare, Dream | Male | Post COVID-19 | 0.197 | 0.047 | **0.000 ***** |
| Insomnia, Sleep | Female | COVID-19 Peak | 0.100 | 0.050 | **0.057 *** |
| Insomnia, Sleep | Male | COVID-19 Peak | -0.016 | 0.052 | 0.759 |
| Insomnia, Sleep | Female | Post COVID-19 | 0.046 | 0.034 | 0.177 |
| Insomnia, Sleep | Male | Post COVID-19 | -0.035 | 0.036 | 0.343 |
| Cold, Fever | Female | COVID-19 Peak | -0.162 | 0.121 | 0.195 |
| Cold, Fever | Male | COVID-19 Peak | -0.047 | 0.080 | 0.568 |
| Cold, Fever | Female | Post COVID-19 | -0.118 | 0.025 | **0.000 ***** |
| Cold, Fever | Male | Post COVID-19 | -0.072 | 0.023 | **0.003 ***** |
| Lose weight | Female | COVID-19 Peak | -0.005 | 0.018 | 0.780 |
| Lose weight | Male | COVID-19 Peak | -0.046 | 0.021 | **0.044 **** |
| Lose weight | Female | Post COVID-19 | 0.032 | 0.018 | **0.088 *** |
| Lose weight | Male | Post COVID-19 | 0.002 | 0.015 | 0.896 |
| Eye | Female | COVID-19 Peak | -0.006 | 0.018 | 0.727 |
| Eye | Male | COVID-19 Peak | -0.035 | 0.017 | **0.045 **** |
| Eye | Female | Post COVID-19 | 0.048 | 0.011 | **0.000 ***** |
| Eye | Male | Post COVID-19 | -0.009 | 0.015 | 0.575 |
| Money | Female | COVID-19 Peak | 0.034 | 0.016 | **0.051 *** |
| Money | Male | COVID-19 Peak | 0.042 | 0.022 | **0.064 *** |
| Money | Female | Post COVID-19 | 0.062 | 0.013 | **0.000 ***** |
| Money | Male | Post COVID-19 | 0.090 | 0.017 | **0.000 ***** |

Note: * P<0.1, ** P<0.05, *** P<0.01.

S6 Table: T-test results of number of posts by 1000 total posts generated by each gender by week.

| Topic | Gender | Period | Coefficient | SE | P-Value |
| --- | --- | --- | --- | --- | --- |
| Nightmare, Dream | Female | COVID-19 Peak | 0.219 | 0.077 | **0.009***** |
| Nightmare, Dream | Male | COVID-19 Peak | 0.096 | 0.057 | 0.106 |
| Nightmare, Dream | Female | Post COVID-19 | 0.215 | 0.037 | **0.000***** |
| Nightmare, Dream | Male | Post COVID-19 | 0.174 | 0.045 | **0.000***** |
| Insomnia, Sleep | Female | COVID-19 Peak | 0.102 | 0.046 | **0.038**** |
| Insomnia, Sleep | Male | COVID-19 Peak | -0.02 | 0.06 | 0.738 |
| Insomnia, Sleep | Female | Post COVID-19 | 0.03 | 0.027 | 0.281 |
| Insomnia, Sleep | Male | Post COVID-19 | -0.041 | 0.033 | 0.233 |
| Cold, Fever | Female | COVID-19 Peak | -0.109 | 0.11 | 0.335 |
| Cold, Fever | Male | COVID-19 Peak | -0.01 | 0.069 | 0.89 |
| Cold, Fever | Female | Post COVID-19 | -0.122 | 0.024 | **0.000***** |
| Cold, Fever | Male | Post COVID-19 | -0.079 | 0.023 | **0.001***** |
| Lose weight | Female | COVID-19 Peak | -0.017 | 0.017 | 0.323 |
| Lose weight | Male | COVID-19 Peak | -0.048 | 0.021 | **0.037**** |
| Lose weight | Female | Post COVID-19 | 0.027 | 0.019 | 0.168 |
| Lose weight | Male | Post COVID-19 | 0.004 | 0.015 | 0.766 |
| Eye | Female | COVID-19 Peak | -0.01 | 0.015 | 0.504 |
| Eye | Male | COVID-19 Peak | -0.026 | 0.017 | 0.145 |
| Eye | Female | Post COVID-19 | 0.04 | 0.012 | **0.002***** |
| Eye | Male | Post COVID-19 | -0.004 | 0.018 | 0.830 |
| Money | Female | COVID-19 Peak | 0.033 | 0.019 | **0.093*** |
| Money | Male | COVID-19 Peak | 0.042 | 0.021 | **0.055*** |
| Money | Female | Post COVID-19 | 0.055 | 0.013 | **0.000***** |
| Money | Male | Post COVID-19 | 0.091 | 0.015 | **0.000***** |

Note: * P<0.1, ** P<0.05, *** P<0.01.

S7 Table: Gender difference in share of cold topic across time

|  | is_cold topic (1 = Yes, 0 = No) |
| --- | --- |
| Female | 0.017*** |
|  | (0.002) |
| COVID-19 | -0.017*** |
|  | (0.002) |
| Female × COVID-19 | -0.009*** |
|  | (0.002) |
|  |  |
| Base share (Male) | 0.034 |
| Age controls | Yes |
| Province FE | Yes |
| Observations | 76,291 |
| Adjusted R2 | 0.005 |

Note: * P<0.1, ** P<0.05, *** P<0.01. We split age in 10-year bins and include a dummy variable for each bin to control the effect of age. We also have included province fixed effects in the regression.


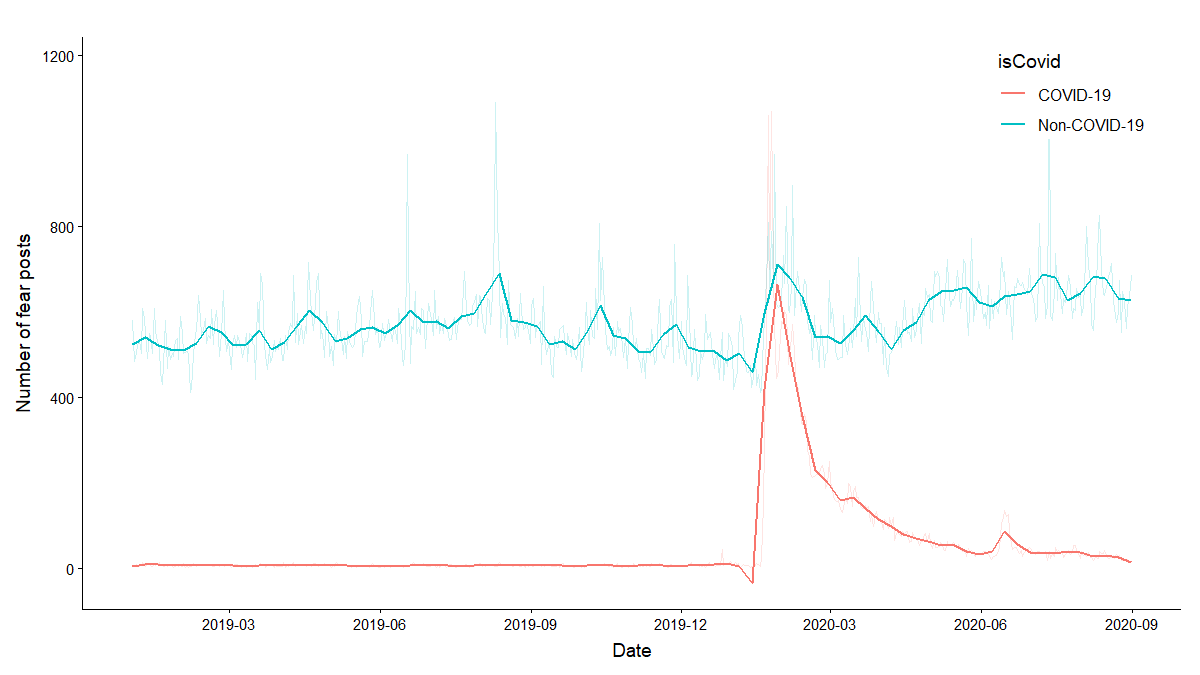


S1 Fig: Temporal trend displays the number of COVID-19 and non-COVID-19 posts per day. The line on the bottom represents COVID-19 related post which started to increase on January 20^th^, reached the peak on January 23^rd^ and gradually decreased after that. The upper line depicts the trend of non-COVID-19 posts, fluctuating around 573 fear posts per day.

A Topics related to COVID-19 B Topics non-related to COVID-19


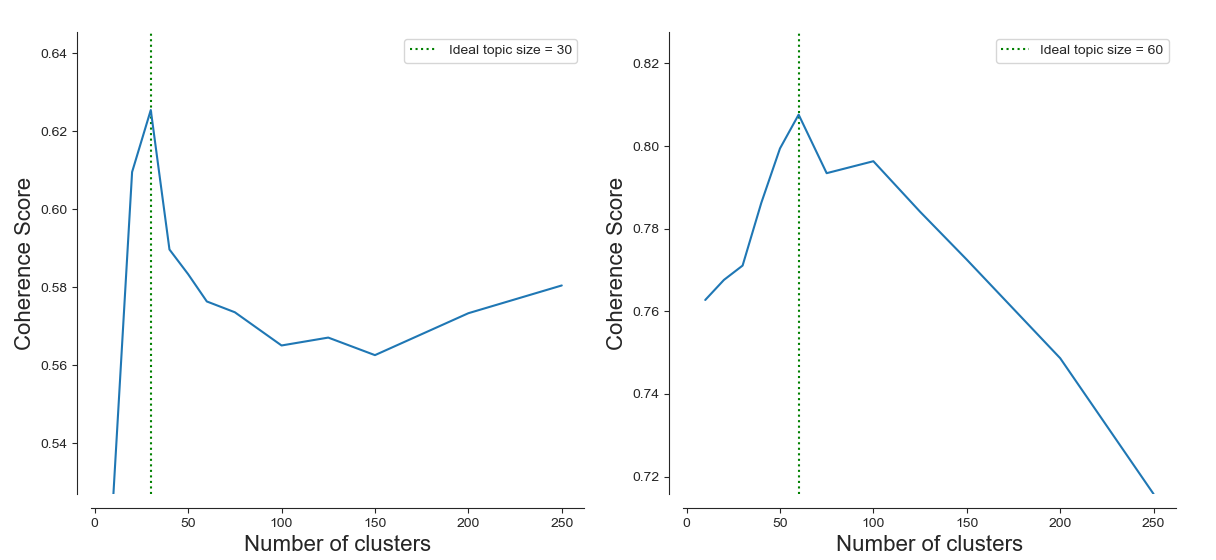


S2 Fig: Coherence score of topic modeling for COVID-19 and non-COVID-19 topics. Coherence score displays the ideal number of clusters for two groups of texts. Panel A (left) shows the highest coherence score for topic clustering on COVID-19 related posts could be achieved when taking 30 as the number of clusters. Panel B (right) shows that setting topic size as 60 would be ideal for non-COVID-19 related posts.


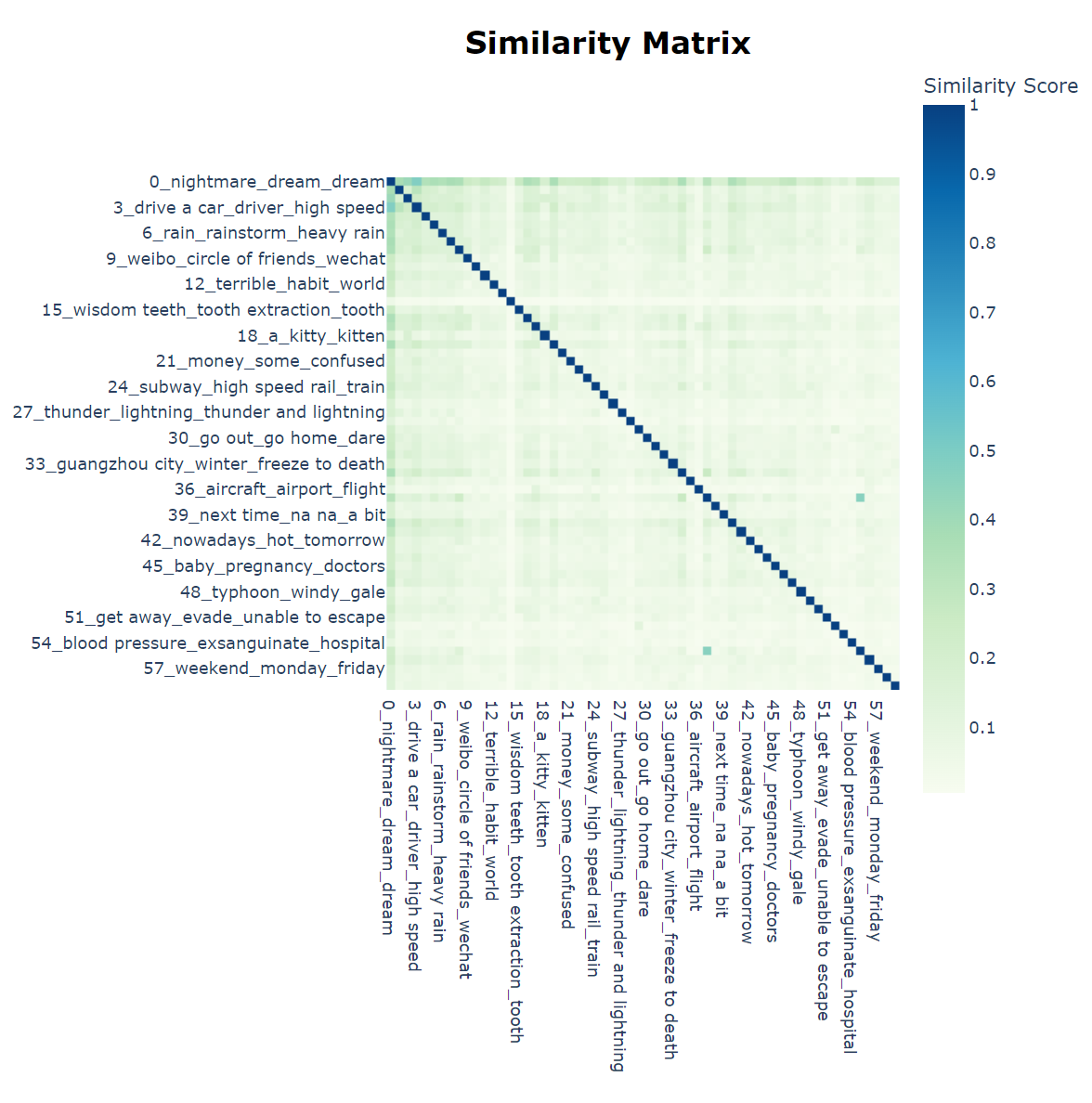


S3 Fig: Similarities between topics. Heat map shows the similarities between topics using the BERTopic embedded function. The label for each topic contains the most informative three keywords extracted using c-TF-IDF as well as the index of the topic. Result suggests that “nightmare” topic shares relevant high similarity with topics including “terror films” and “heavy rains”; while “insomnia” topic is closely associated with “start of the school” and “drink alcohol”.


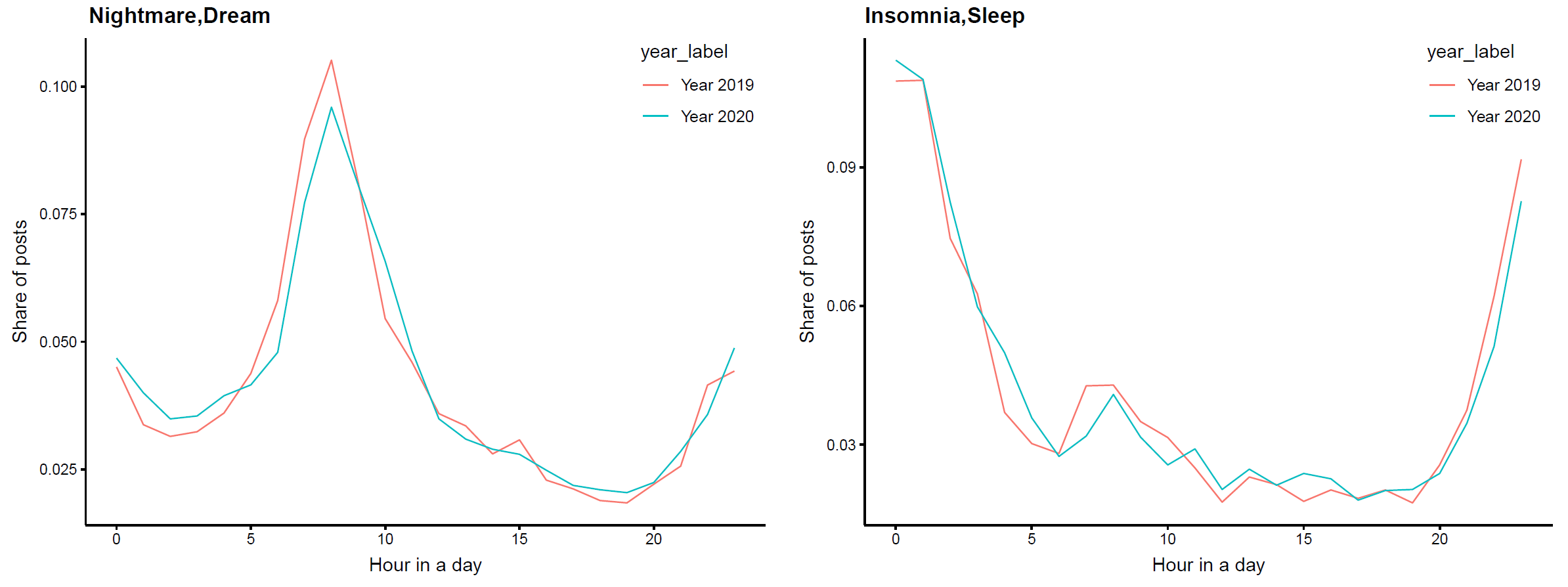


S4 Fig: Temporal trend of “Nightmare” and “Insomnia” topics. Line graphs show the temporal distribution of posting a specific topic within a day by year. Neither the nightmare topic nor insomnia topic changes significantly when comparing the years 2019 and 2020, indicating the usage of the word remains constant.


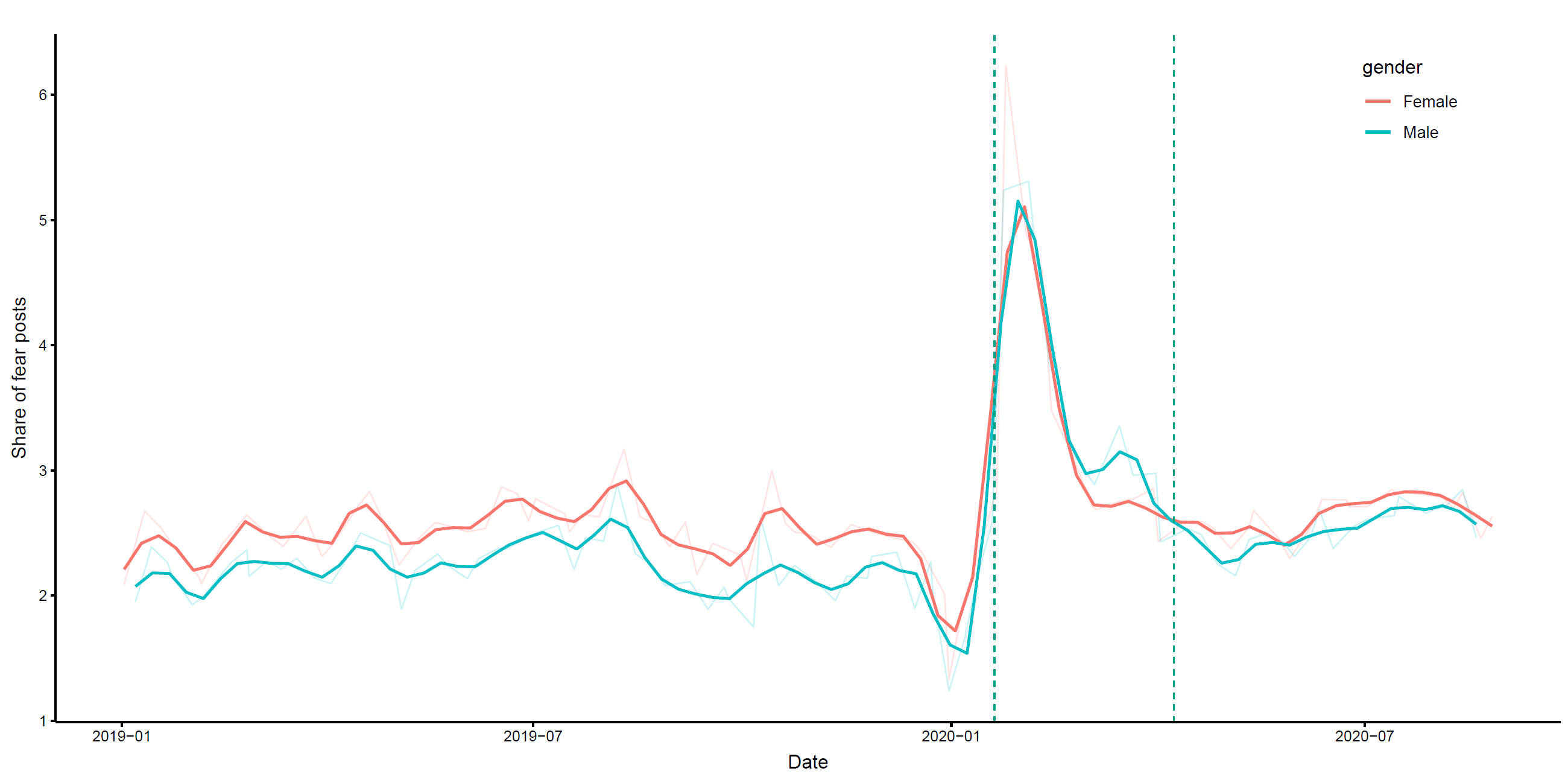


S5 Fig: Line graph shows the share of posts having fear as the dominant emotion by gender.


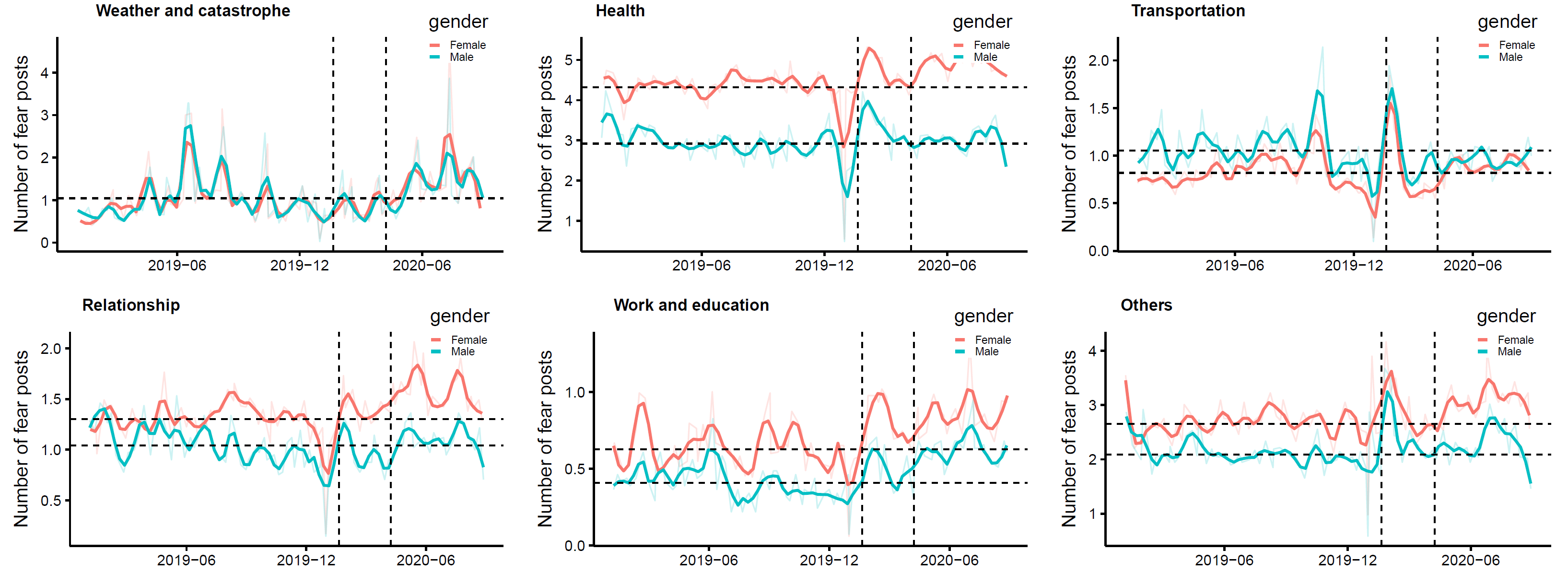


S6 Fig: Line graphs show the weekly number of posts in six general aspects including “Weather and Catastrophe”, “Health”, “Transportation”, “Relationship”, “Work and Education” and “Others”
